# The Presence of HLA-E-Restricted, CMV-Specific CD8^+^ T Cells in the Blood of Lung Transplant Recipients Correlates with Chronic Allograft Rejection

**DOI:** 10.1371/journal.pone.0135972

**Published:** 2015-08-24

**Authors:** Lucy C. Sullivan, Glen P. Westall, Jacqueline M. L. Widjaja, Nicole A. Mifsud, Thi H. O. Nguyen, Aislin C. Meehan, Tom C. Kotsimbos, Andrew G. Brooks

**Affiliations:** 1 Department of Microbiology and Immunology, Peter Doherty Institute for Infection and Immunity, The University of Melbourne, Parkville, Victoria, Australia; 2 Department of Medicine, Monash University, Central Clinical School, The Alfred Centre, Commercial Road, Melbourne, Victoria, Australia; 3 Department of Allergy, Immunology and Respiratory Medicine, The Alfred Hospital, Commercial Road, Melbourne, Victoria, Australia; Cedars-Sinai Medical Center, UNITED STATES

## Abstract

The human cytomegalovirus (CMV) immune evasion protein, UL40, shares an identical peptide sequence with that found in the leader sequence of many human leukocyte antigen (HLA)-C alleles and when complexed with HLA-E, can modulate NK cell functions via interactions with the CD94-NKG2 receptors. However the UL40-derived sequence can also be immunogenic, eliciting robust CD8+ T cell responses. In the setting of solid organ transplantation these T cells may not only be involved in antiviral immunity but also can potentially contribute to allograft rejection when the UL40 epitope is also present in allograft-encoded HLA. Here we assessed 15 bilateral lung transplant recipients for the presence of HLA-E-restricted UL40 specific T cells by tetramer staining of peripheral blood mononuclear cells (PBMC). UL40-specific T cells were observed in 7 patients post-transplant however the magnitude of the response varied significantly between patients. Moreover, unlike healthy CMV seropositive individuals, longitudinal analyses revealed that proportions of such T cells fluctuated markedly. Nine patients experienced low-grade acute cellular rejection, of which 6 also demonstrated UL40-specific T cells. Furthermore, the presence of UL40-specific CD8^+^ T cells in the blood was significantly associated with allograft dysfunction, which manifested as Bronchiolitis Obliterans Syndrome (BOS). Therefore, this study suggests that minor histocompatibility antigens presented by HLA-E can represent an additional risk factor following lung transplantation.

## Introduction

The role of non-classical major histocompatibility (MHC) class I molecules (class Ib) such as human leukocyte antigen (HLA)-E in adaptive immunity are only just beginning to be understood. Unlike the highly polymorphic classical HLA-A,-B and-C molecules (MHC class Ia), HLA-E is far less polymorphic with 15 alleles described to date of which only two are common and that differ from each other by a single amino acid [1]. The best-characterised function of HLA-E is to act as a ligand for the CD94-NKG2 receptors expressed on natural killer (NK) and T cells [2,3]. Recognition of HLA-E by CD94-NKG2A transduces inhibitory signals thereby blocking NK cell activation, whereas engagement of CD94-NKG2C can activate NK cells [2,4].

NK cell recognition of HLA-E expressed on healthy cells is acutely dependent on the presence of conserved peptides found in the leader sequence of MHC class Ia molecules [2,5,6]. Consequently, it has been hypothesised that the major function of HLA-E is to allow NK cells to broadly monitor the expression of diverse MHC-I molecules since the acquisition of these peptides by HLA-E and their subsequent transport to the cell surface is also dependent on the integrity of antigen processing machinery [3]. Hence any loss of HLA-E/class I-derived peptide complexes that may occur as a result of transformation or viral infection can promote target cell lysis by CD94-NKG2A^+^ NK cells.

While HLA-E acts as a ligand for these germline-encoded receptors, it can also present pathogen-derived antigens for T cell receptor (TCR)-dependent recognition by CD8^+^ T cells. Indeed, HLA-E-restricted T cells may play a significant role in immunity to a number of human pathogens including *Salmonella typhi*, *Mycobacterium tuberculosis* and human cytomegalovirus (CMV) [7–11]. CMV is a large DNA virus that establishes a lifelong asymptomatic infection in healthy individuals. Nevertheless, CMV reactivation and/or infection causing significant morbidity can occur in immunocompromised individuals, such as those with immune deficiencies (HIV) or immunosuppressed patients following transplantation.

CMV has evolved a number of mechanisms to evade the immune system, including one that manipulates the expression of HLA-E [12]. The CMV glycoprotein UL40 typically contains a sequence that is identical to the HLA-E-binding peptide found in many HLA-C alleles. Hence, the expression of UL40 can enhance cell surface expression of HLA-E and protect cells from NK cell-mediated lysis [12,13], despite the fact that CMV infection can also downregulate the expression of classical MHC-I molecules.

However, Pietra *et al*. demonstrated that HLA-E could be recognised by a population of CD8^+^ T cells, termed NK-CTL [14]. Subsequent analyses showed that NK-CTL were specific for the UL40-encoded mimic of the MHC-I leader sequence peptide, VMAPRTLIL [15] and hence were actually UL40-specific T cells. Analyses of the fine specificity of UL40-specific T cell clones from multiple donors suggested that HLA genotype was critical in determining whether or not such cells were present in a given individual, as well as impacting the fine specificity of any such clones. Typically, UL40-specific T cells were not isolated from individuals that possessed HLA-C alleles that contained the sequence VMAPRTLIL suggesting that HLA-driven expression of these peptides derived from these alleles induced a degree of tolerance [15]. In contrast, where both HLA-C alleles lacked this sequence (eg HLA-C*02,-C*07,-C*15) and the donor was CMV seropositive, UL40-specific T cells were detected in the blood. This data suggested that the HLA class I genotype, through the provision of HLA-E binding peptides that were capable of negatively selecting potentially autoreactive cells, was a key determinant of whether HLA-E-restricted UL40-specific T cells were present in the repertoire.

In the setting of transplantation, the capacity to elicit HLA-E-restricted, UL40-specific T cells may be particularly important. Specifically such cells may create an additional rejection risk as they have been shown to lyse a large array of allogeneic target cells [16]. Consequently, *in vivo*, these cells have the potential to contribute to graft rejection through recognition of allogeneic MHC-I leader sequences that are identical to that in UL40. Indeed, the presence of a UL40-specific T cell population as a result of CMV infection prior to transplantation will essentially constitute an expanded pool of allograft specific T cells. Therefore, this system represents a unique opportunity to assess the effect of cross-reactive antiviral T cells in a transplantation setting, where the identity of the peptide antigen has been clearly defined. Hence, a better understanding of UL40-specific T cells and the impact of HLA genotype on their ability to recognize HLA-E/MHC-I leader sequence peptides may determine whether these minor antigens represent an additional risk factor following solid organ transplantation.

Here we assessed 15 bilateral lung transplantation recipients with appropriate HLA genetics (HLA-C*02,-C*07,-C*15) for the presence of UL40-specific T cells in peripheral blood mononuclear cells (PBMC) within the first 12 months following transplantation. Fourteen of the 15 recipients received an HLA-C mismatched allograft. We hypothesised that the presence of allogeneic peptides derived from MHC-I leader sequences expressed by cells within the lung allograft may result in the activation and expansion of UL40-specific T cells that ultimately contributes to either acute and/or chronic graft rejection.

## Materials and Methods

### Ethics statement

All patients and controls gave written informed consent and the study was approved by The Alfred Hospital Ethics Committee (Project 175/02) and The University of Melbourne Human Research Ethics Committee (Project 0709337). None of the transplant donors were from a vulnerable population and all donors or next of kin provided written informed consent that was freely given.

### Healthy non-transplant donors

PBMC were isolated from the blood of healthy non-transplant donors by Ficoll-Paque (GE Healthcare) density gradient. Individuals were typed for HLA-C by the Victorian Transplantation and Immunogenetics Service (VTIS).

### Clinical Cohort

Over a period of 4 years, 118 patients receiving a bilateral lung transplant at The Alfred Hospital were recruited to the study. HLA-C typed by VTIS. Of these, 25 individuals (21.1%) possessed an HLA-C type permissive for the expansion of UL40-specific T cells (HLA-C*02,-C*07,-C*15). The final clinical cohort consisted of 15 lung transplant recipients that were available for follow-up at The Alfred with PBMC samples obtainable to 12 months post-transplant (Table 1). All patients received a standard triple immunosuppressant regimen consisting of prednisolone, azathioprine and cyclosporine A or tacrolimus. All patients at risk of CMV reactivation (either donor- or recipient-positive CMV serostatus) received antiviral prophylaxis for 5 months. Surveillance post-transplant bronchoscopies were performed at 2 weeks and 1, 2, 3, 6 and 12 months, or if clinically indicated with peripheral blood, bronchoalveolar lavage (BAL) and transbronchial biopsies being collected at these time points. Clinical variables analysed included acute cellular rejection (ACR), infection (viral and bacterial), BOS and mortality. ACR and CMV pneumonitis was diagnosed by histopathological changes identified on transbronchial biopsy specimens and/or CMV pneumonitis according to standard histopathological criteria [17,18]. CMV infection was defined by detection of CMV DNA in BAL fluid by COBAS Amplicor CMV monitor test (Roche Diagnostic Systems, NSW, Australia). BOS was diagnosed on physiologic criteria of a sustained and irreversible reduction in forced expiratory volume (FEV1) below 80% of personal best achieved post-transplant in the absence of any other identifiable cause. All patients were monitored for a minimum of 4 years post-transplant [17]. Clinically stable patients were defined as being alive and free from BOS 12 months post-transplant.

**Table 1:** Lung transplant recipient demographics.

Transplant Recipient #	Patient age at transplant	Recipient HLA-A	Recipient HLA-B	Recipient HLA-C	Donor HLA-A	Donor HLA-B	Donor HLA-C	Donor (D) Recipient (R) CMV serostatus
1	56	24,68	18,44	2,15	2,33	60,65	3,8	D+/R+
2	29	1,3	7,8	7	2,32	18,62	3,7	D+/R+
3	32	1	8	7	3,31	7,60	3,7	D-/R+
4	54	2,3	7	7	1,3	8,39	7	D+/R+
5	19	2,3	7,27	2,7	1,31	57,60	3,6	D+/R-
6	51	1,2	7,8	7	2,34	13,56	4,7	D+/R+
7	57	1	7,8	7	1,2	8,62	3,7	D-/R+
8	64	2,24	18,44	7	2,11	39,60	3,7	D+/R+
9	22	1,3	7,8	7	11,30	13,51	6,14	D-/R+
10	35	2,3	7,8	7	11,33	27,58	3, 12	D+/R+
11	56	1	8	7	3, 11	55,62	3, 3	D-/R+
12	56	2,11	18	7	2, 11	18,44	5, 7	D+/R-
13	58	1,3	7,8	7	11, 30	35,51	4, 16	D+/R+
14	65	1,32	8,70	7	2, 3	7, 62	3, 7	D+/R+
15	47	2	7	7	1, 3	7, 57	6, 7	D+/R+
Mean (SE)	46.7 (3.9)							

### Samples

Peripheral blood samples were collected to at least 12 months post-transplant and PBMC were isolated using Ficoll-Paque and cryopreserved for subsequent FACS analysis. Longitudinal samples from a single recipient were analysed on the same day. One healthy non-transplant donor in which the presence of an HLA-E restricted UL40-specific T cell subset had been previously confirmed [19] was used as a positive control in each analysis.

### Flow cytometric analysis

HLA-E tetramers bound to the leader peptide derived from HLA-C*03/UL40 (sequence VMAPRTLIL, HLA-E^VMAPRTLIL^) or HLA-G (VMAPRTLFL, HLA-E^VMAPRTLFL^) were generated as described previously [20]. Frozen PBMC samples were thawed rapidly and checked for cell viability with trypan blue exclusion cell counting. Each sample was stained with either HLA-E^VMAPRTLIL^ or HLA-E^VMAPRTLFL^ with or without blocking antibodies to CD94 (supernatant from the XA185 hybridoma in combination with anti-CD94 (clone HP-3B1 (Beckman Coulter)) in order to prevent the tetramer binding to CD94-NKG2 receptors expressed on T cells. Fluorochrome-conjugated antibodies were added: CD27-FITC (BD Biosciences), CD8β-PE (Beckman Coulter) (or CD3-PECy7 and CD8α-APC-Cy7, BD Biosciences) and CD45RA-Alexa 700 (BD Biosciences). Flow cytometric analysis was performed using a Becton Dickinson FACSCanto II. Samples were analysed using FlowJo software (Treestar, San Carlos, USA).

### Statistical analysis

Two-tailed Fisher’s exact tests (GraphPad) were used to compute p values from contingency tables.

## Results

### UL40-specific T cells can be detected in the peripheral blood of healthy non-transplant donors

**Fig 1:**
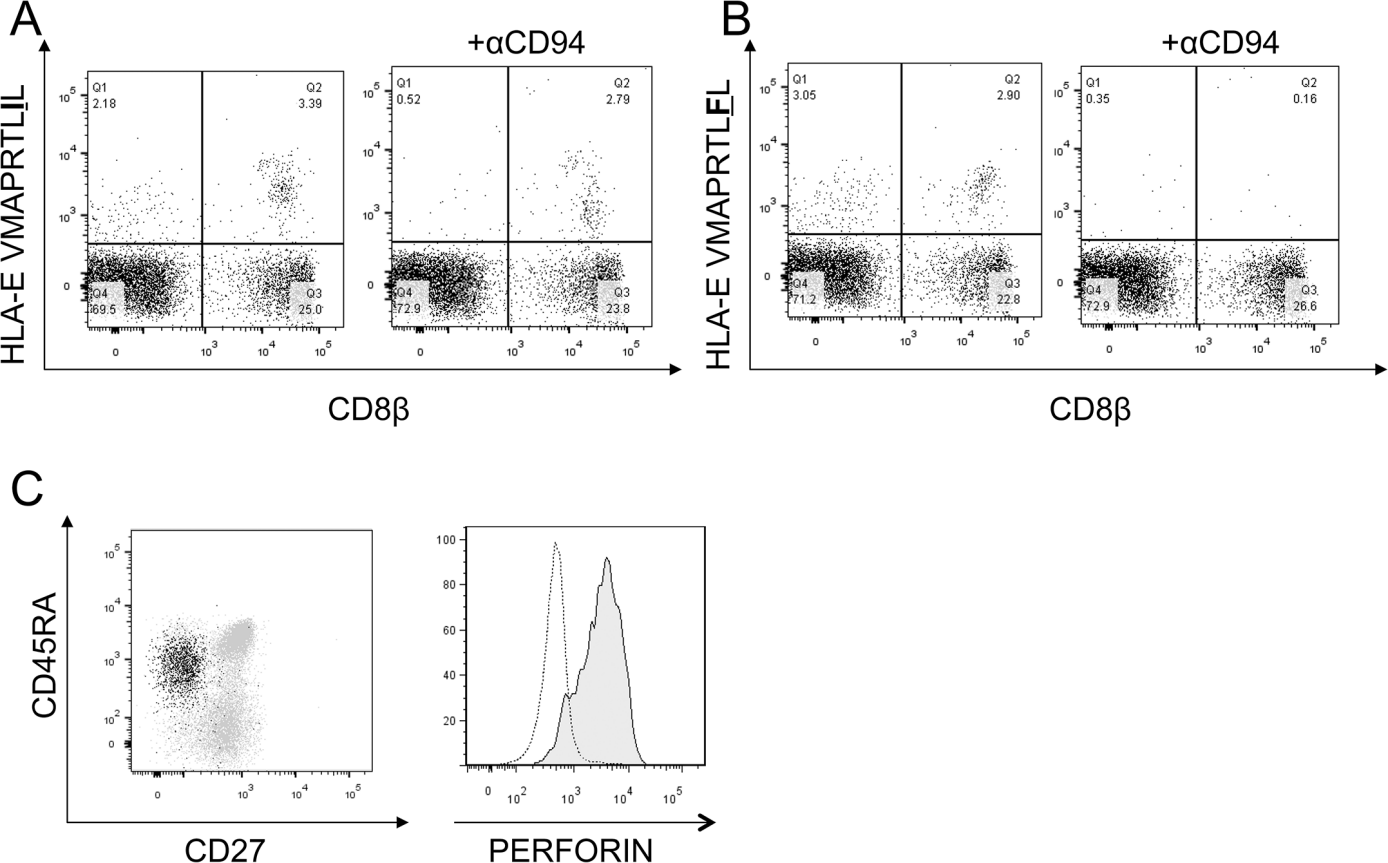
UL40-specific T cells isolated from a healthy donor have an effector memory phenotype. Peripheral blood mononuclear cells (PBMC) were isolated from a healthy donor and were stained with HLA-E tetramers refolded with either the leader sequence from HLA-C*03 (HLA-E-VMAPRTL**I**L, A) or HLA-G (HLA-E-VMAPRTL**F**L, B) in the presence (+αCD94) or absence of a blocking antibody to CD94. Cells were also stained with antibodies to CD8β, CD27 and CD45RA (C left panel: tetramer positive, CD8β+ cells: black, tetramer negative CD8β+: gray) before permeabilising and staining with an antibody to perforin (C right panel, tetramer positive, CD8β+ cells: solid histogram, tetramer negative CD8β+: dotted line). Numbers in each quadrant reflect percentages of live lymphocytes.

To validate our capacity to identify HLA-E restricted UL40-specific T cells directly *ex vivo*, PBMC from healthy CMV seropositive non-transplant donors with HLA-I genotypes that did not encode the VMAPRTLIL sequence (eg HLA-C*02/HLA-C*07) were stained with anti-CD8β together with HLA-E tetramers. As expected HLA-E tetramer complexed to either VMAPRTLIL or VMPARTLFL bound to a large population of CD8β^-^ cells together with another distinct population of CD8β^+^ cells since CD94-NKG2 receptors are widely expressed on both NK cells and CD8^+^ T cells. Consequently, to identify cells that bound tetramer via clonotypic TCRs rather than the CD94-NKG2 receptors, tetramer staining was performed in the presence of a blocking CD94-specific antibody. The efficacy of this antibody in blocking this interaction is evident in the inhibition of tetramer staining to CD8β^-^ cells in the upper left quadrant of the dot plots (Fig 1). Critically, in the presence of blocking CD94-specific mAb, a distinct population of tetramer positive, CD8β+ cells was evident following staining with HLA-E^VMAPRTLIL^ but not HLA-E^VMAPRTLFL^ indicating the specific binding of the HLA-E^VMAPRTLIL^ tetramer to the TCR of UL40-specific T cells (Fig 1A and 1B). Co-staining with mAbs specific for CD45RA, CD27 and perforin showed that the majority of these HLA-E^VMAPRTLIL^ tetramer+ cells were CD27-CD45RA+ and expressed high levels of perforin relative to tetramer negative CD8β^+^ cells suggesting that they were effector memory cells (Fig 1C). Similar patterns of expression were seen on at least 3 donors albeit that the proportion of UL40-specific cells varied markedly (data not shown).

### UL40-specific CD8 T cells are present in the peripheral blood of lung transplant recipients

Sequencing of UL40 genes from clinical samples has shown that most CMV isolates possess a UL40 gene that encodes the VMAPRTLIL sequence identical to that present in most HLA-C allotypes [21,22]. Consequently we hypothesised that transplantation of allogeneic lungs from donors who possess this HLA-C-encoded sequence into recipients who lack this sequence may constitute a risk factor for alloreactivity. We determined the HLA-C genotype of 118 patients and found 25 who lacked HLA-encoded VMAPRTLIL sequences. Longitudinal PBMC samples were available for 15 of these recipients within 12 months post-transplant. Of these 15 patients, 14 received an HLA-C mismatched organ. The presence of UL40-specific CD8^+^ T cells in the PBMC of these patients was then assessed using an HLA-E^VMAPRTLIL^ tetramer in conjunction with a blocking CD94-specific mAb as described above.

**Fig 2:**
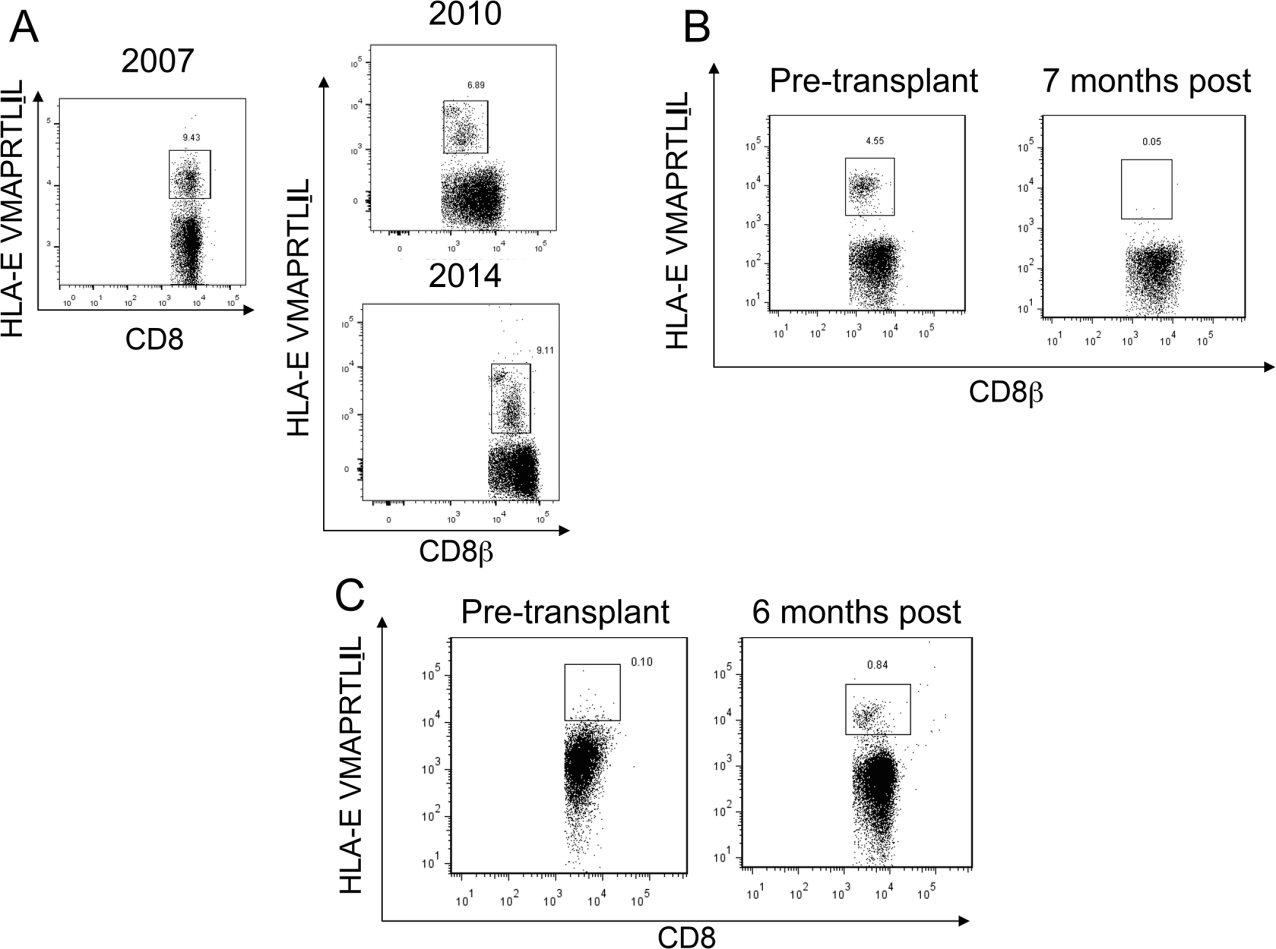
Appearance of UL40 specific T cells differs in lung transplant recipients to a healthy non-transplant donor. The percentage of UL40-specific T cells remains stable in a healthy non-transplant donor over a seven-year period (A). UL40-specific T cells are present in the blood of LTR #10 prior to transplant but were not observed at any other sampling time point post transplantation (B). UL40-specific T cells are not present in the blood of LTR #2 prior to transplant but appear in the blood 6 months post-transplant, coincident with AR1 (C). Plots showing CD8β are gated only on lymphocytes, whereas those showing CD8 are gated on CD3 positive lymphocytes. Numbers show UL40-specific T cells as a percentage of total CD8+ T cells.

**Fig 3:**
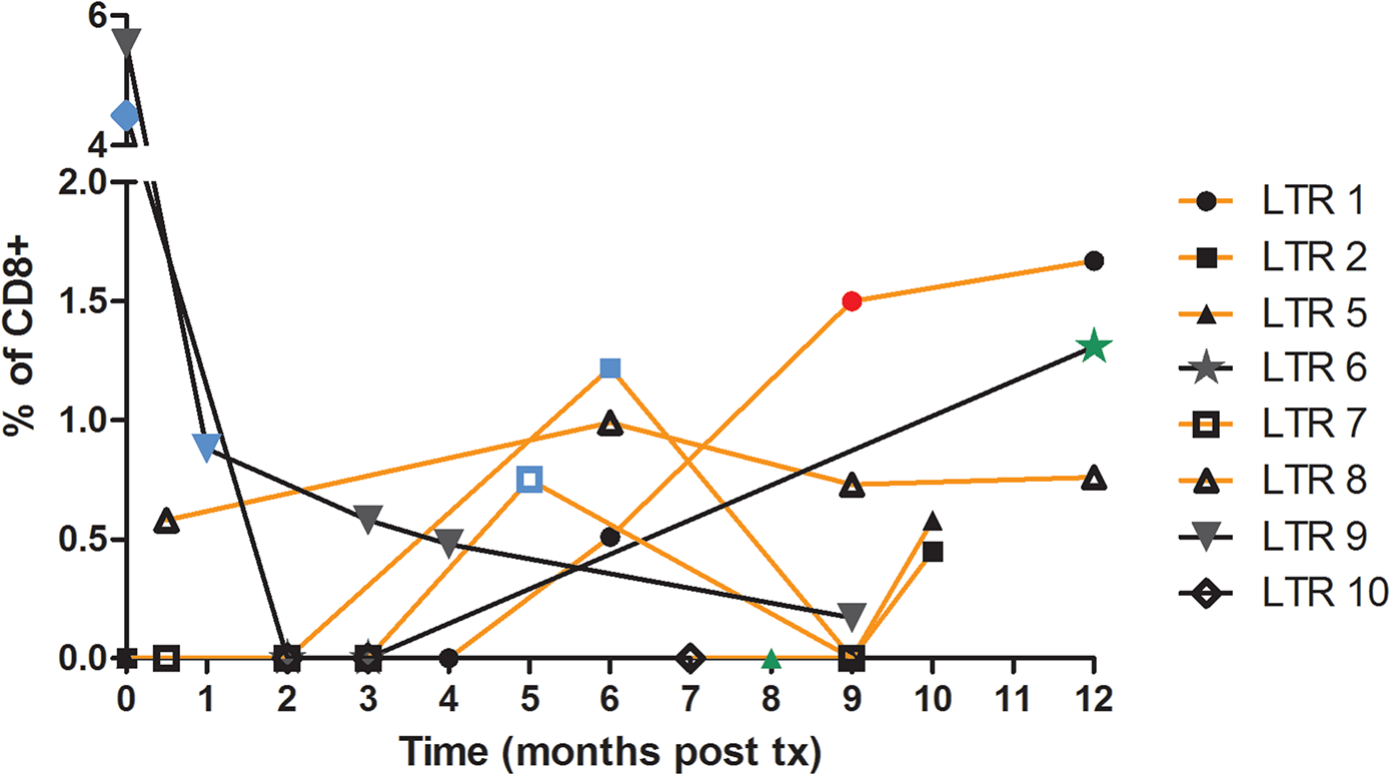
UL40-specific T cell dynamics correspond to clinical events in lung transplant recipients. Longitudinal samples were collected from 15 LTR for 12 months post-transplant and PBMC were stained with HLA-E tetramers refolded with the UL40 epitope in the presence of blocking CD94 mAb. Graph shows UL40-specific T cells as a percentage of total CD8+ T cells for 8 patients where a population of UL40-specific T cells was observed for at least one time point (LTR #10 only observable pre-transplant). Blue symbols: Acute rejection (AR) episode as determined by transbronchial biopsy; Green symbols: Cytomegalovirus (CMV) reactivation as determined by PCR in bronchoalveolar lavage; Red symbol: concurrent AR and CMV; Orange lines: LTR who developed chronic allograft dysfunction, manifested as Bronchiolitis Obliterans Syndrome (BOS).

Pre-transplant samples were available for analysis in 9 CMV seropositive patients, with UL40-specific CD8^+^ T cells detected in only 2 patients (LTR #9, #10; Figs 2 and 3). Following transplantation and induction of immunosuppression, the frequency of UL40-specific CD8^+^ T cells (as a proportion of the total CD8^+^ T cell pool) in these two patients decreased significantly (LTR #9, 5.56% to 0.55%; LTR #10, 4.55% to 0.05%, Fig 2B). This was in contrast to that observed in a healthy non-transplant donor where the proportion of UL40-specific T cells remained relatively stable, in one case for a 7-year period (Fig 2A). Whilst two patients were positive for UL40-specific CD8^+^ T cells pre-transplant (Fig 3), following transplantation the proportion of patients in whom UL40-specific CD8 T cells were identified was considerably higher, being observed in seven patients (UL40-specific T cells were observed in pre- and post-transplant samples in LTR#9). Of interest, the first *de novo* appearance of UL40-specific CD8^+^ T cells in PBMC isolated from five patients (LTRs #1, #2, #5, #6, #7) was not early post-transplant during the period of maximal immunosuppression, but rather beyond 5 months (Figs 2C and 3), also corresponding to the cessation of routine antiviral prophylaxis.

### Appearance of UL40-specific T cells and correlation with clinical events

Longitudinal changes in the numbers of UL40-specific T cells may primarily reflect a role in antiviral immunity. Viewed from this perspective, increased numbers of UL40-specific T cells in PBMC samples would be expected to reflect CMV reactivation. All patients were at risk of CMV reactivation (donor or recipient CMV seropositive) and received antiviral prophylaxis for 5 months following lung transplantation. Six patients developed CMV reactivation beyond 6 months post-lung transplant (Table 2). Of these, UL40-specific T cells were identified in 3 donors at time points, concomitant (#1 and #6) or just following (#5) CMV reactivation at 9 (#1), 10 (#5; CMV at 7 and 8 months) and 12 months (#6) post-transplant. Two patients (#11, #13) developed CMV reactivation at time points after 12 months post-transplant, and PBMC samples were not available for analysis beyond this time to assess whether UL40-specific CD8^+^ T cells developed at a later point in response to the CMV reactivation. However, there was no association between the presence of UL40-specific T cells in the blood with the incidence of CMV reactivation (Table 3).

**Table 2:** Clinical Complications post Lung Transplant.

Tx#	UL40 Specific T cells Post Tx*	BOS	Months to BOS	AR>/ = 1	AR>/2	Time to first AR	CMV	Days to CMV
1	yes	yes	17	yes	yes	284	yes	284
2	yes	yes	24	yes	no	191	no	—
3	no	no		yes	yes	14	no	—
4	no	no		yes	no	13	no	—
5	yes	yes	8	no	no	—	yes	193
6	yes	no		yes	yes	55	yes	355
7	yes	yes	11	yes	yes	40	no	—
8	yes	yes	60	yes	no	30	no	—
9	yes	no		yes	yes	14	no	—
10	no	no		no	no	—	yes	270
11	no	no		no	no	—	yes	378
12	no	no		no	no	—	no	—
13	no	no		yes	yes	26	yes	355
14	no	no		no	no	—	no	—
15	no	no		no	no	—	no	—

* Only patients who demonstrated UL40-specific T cells in the blood **post** transplant are shown on this table.

**Table 3:** Post-transplant* UL40-specific CD8+ T cells: Clinical associations.

	UL40-specific CD8+ T cell-ve	UL40-specific CD8+ T cell +ve	p-value#
Acute Cellular Rejection			0.11
Negative	5	1	
Positive	3	6	
Bronchiolitis Obliterans Syndrome			0.007
Negative	8	2	
Positive	0	5	
CMV Reactivation			1
Negative	5	4	
Positive	3	3	

*Only patients who demonstrated UL40-specific T cells in the blood **post** transplant are shown on this table.

# p-values calculated by two-tailed Fisher’s exact test

The similarity between the UL40-encoded sequence and peptides corresponding to the leader sequence of classical HLA-I proteins also means that UL40-specific cells may also be allograft reactive. Indeed within this cohort, there was sequence identity between peptides derived from allograft-encoded HLA-C and the canonical UL40 sequence in all but one patient. Interestingly, no UL40-specific T cells were observed in this patient who received an HLA-C*07 matched allograft (LTR #4). In the remaining patients, the appearance of UL40-specific T cells may correlate with clinical outcomes, and in particular, to episodes of either acute (ACR) or chronic lung allograft dysfunction, as manifested by BOS. Therefore, we assessed whether the presence of UL40-specific T cells correlated with increased frequency or severity of ACR as assessed by routine lung biopsy. Overall 9/15 patients experienced low-grade (A1) rejection, of which 6 also demonstrated UL40-specific T cells (Table 2, Fig 2). However this value was not significantly different from LTR where UL40-specific T cells were not observed (Table 3). Chronic lung allograft dysfunction (BOS) clinically manifests as a drop in spirometry, with the major risk factor being ongoing alloreactivity. In this cohort, the development of BOS was not associated with age at the time of transplant (stratified as less than or more than 50 years at the time of transplant), nor the number of HLA-I mismatches (stratified as 2–4 or 5–6 mismatches, data not shown). However, by the census date, BOS had developed in 5 patients, all of whom had developed UL40-specific T cells following transplantation. Indeed, the presence of UL40-specific T cells was significantly associated with the development of BOS albeit that the cohort size was still relatively small (p = 0.007, Table 3).

## Discussion

The high frequency of HLA-E-restricted, UL40-specific T cells in the circulation of several unrelated healthy donors suggests these T cells may play a significant role in CMV immunity. Hence, in lung transplant recipients with permissive HLA genetics (i.e. individuals that lack HLA sequences corresponding to those in UL40), it is possible that these T cells may also help control post-transplant CMV infection or reactivation. In patients who demonstrated *de novo* UL40-specific T cells, these cells were first identified between 6 and 12 months post-transplant, a period that coincides with the cessation of antiviral prophylaxis and greatest risk for CMV reactivation (both clinical and subclinical), suggesting antigen-driven expansion. Nevertheless, the frequency of UL40-specific T cells in the blood did not always increase coincident with CMV reactivation. This lack of correlation is not completely unexpected as the capacity to detect CMV replication is imperfect. Indeed, subclinical viral replication may well remain undetected as a result of sampling issues in obtaining BAL fluids. Similarly, it is also possible that activated UL40-specific T cells preferentially traffic to the allograft resulting in only transient perturbations in the numbers of such cells circulating in the blood. Additionally, several studies highlight the effect of immunodominance where the presence of certain HLA-I alleles impacts the capacity to elicit a response restricted by another allele. For example in individuals that possess HLA-B*07:02, T cells restricted by this HLA-I allele are expanded in response to CMV (specific to a pp65 derived epitope), apparently at the expense of T cells restricted by other HLA alleles such as HLA-A*02:01 [23]. Hence in some individuals, CMV reactivation may induce the expansion of T cell populations restricted by classical HLA-I alleles in preference to HLA-E restricted, UL40-specific T cells.

In addition to their role in CMV immunity, HLA-E restricted, UL40-specific CD8^+^ T cells were originally shown to lyse a large array of allogeneic target cells. Despite this broad pattern of alloreactivity, their potential to impact on transplantation outcome has not been well characterised. Indeed, their potential to recognise cells infected with CMV (both patient and graft-derived) in addition to uninfected allograft tissue suggests that they may both help limit viral reactivation but also directly damage the allograft.

We were unable to demonstrate a direct association between biopsy-proven episodes of ACR and the expansion of UL40-specific CD8^+^ T cells in the blood of lung transplant recipients. Nevertheless, this does not exclude a role for such cells in ACR as the cohort size was relatively small. Further, analysis of lung biopsies is a relatively crude method for defining ACR. Biopsies are taken infrequently, and the assessment of ACR is limited to that time point. Moreover, as transbronchial biopsies are themselves very small (1–2mm), ACR processes may well be present in other parts of the lung, but are missed due to sampling limitations.

It is also possible that the graft-reactive UL40-specific T cells are largely removed from the circulation, preferentially localising to the allograft with only a small proportion evident in PBMC samples. Furthermore, as HLA-E is typically expressed at significantly lower levels than classical HLA, it is possible that the amount of presented antigen is not sufficient to activate UL40-restricted T cells in the absence of another signal, necessitating the need for factors such as co-stimulatory molecules or inflammatory cytokines. These signals may be provided by transplant-associated inflammation such as surgical trauma, ischaemia-reperfusion injury or viral infection.

Numerous studies have linked reactivation of CMV with organ rejection [24]. In this context CMV reactivation may lead to the recruitment and activation of UL40-specific T cells, which may in turn promote to organ rejection due to their direct recognition of the mismatched graft. Importantly, while there was no association between the presence of UL40-specific T cells and ACR, there was an association with the development of BOS, which represents chronic lung allograft dysfunction. Therefore, the complex interplay between CMV reactivation and patterns of alloreactivity may indeed indicate a role for HLA-E restricted UL40-specific T cells in solid organ rejection but will require studies with significantly larger cohorts to confirm.
